# Visual and treatment outcomes of tubercular uveitis: a prospective case series from a referral hospital in Pakistan

**DOI:** 10.1186/s13104-019-4432-8

**Published:** 2019-07-15

**Authors:** Muhammad Ishaq Ghauri, Nousheen Iqbal, Syeda Urooj Riaz, Muhammad Irfan, Ajeet Kumar, Muhamad Shariq Mukarram

**Affiliations:** 1 Department of Medicine, Jinnah Medical College Hospital, Karachi, Pakistan; 2 Section of Pulmonary and Critical Care Medicine, Department of Medicine, Aga Khan University, Stadium Road, Karachi, Pakistan

**Keywords:** Ocular TB, Uveitis, Anti tuberculous treatment, Tuberculous uveitis

## Abstract

**Objective:**

Pakistan is the fifth highest TB burden country. Tuberculous uveitis (TbU) is a form of extrapulmonary TB, that is not uncommon in high burden country but very limited data is available on its outcome. The aim of the study is to assess the outcome of TbU with anti-tuberculous treatment (ATT).

**Results:**

A prospective study was conducted at Jinnah Medical College Hospital (JMCH) Karachi, Pakistan from July to December 2017. Patients with suspected TbU were started on standard ATT chemotherapy for 12 months. Their response was assessed via slit lamp examination and visual acuity at 1, 3, 6 and 12 months of treatment. Forty patients with probable TbU were treated with ATT, mean age was 36 ± 3 years and 24 (60%) were females. Around 26 (65%) had Monteux test of 15 mm or more. History of TB contact was positive in 24 (60%) and 12 (30%) had previous history of TB. All patients complained for blurring of vision and floaters. Posterior uveitis seen in 36 (90%) of patients. Complete response achieved in 32 (80%) after ATT while 6 (14%) had changed in inflammation and 2 (6%) had no benefit.

## Introduction

Tuberculosis (Tb) is a leading cause of mortality and morbidity worldwide, especially in South East Asia regions which carries high burden of Tb. Tb can involve any part of the body, it causes both pulmonary and extrapulmonary diseases. Tb is preventable and completely curable disease despite of this an estimated 1.3 million deaths among HIV negative caused in 2017 [1]. Pakistan is the fifth highest Tb burden country worldwide.

Tuberculous uveitis (TbU) is a rare entity of extra pulmonary Tb where uveal track involvement is common. It causes granulomatous anterior uveitis, disseminated choroiditis with vitritis, and cystoid macular edema [2]. TbU sometimes manifest with non-specific features ranging from non-granulomatous anterior uveitis to occlusive retinal vasculitis [3]. Prevalence of TbU is variable in different parts of world, being more common in south East Asia. A study from Pakistan showed that granulomatous diseases are the frequent cause of uveitis likely because of high burden of Tb [4].

Tuberculous uveitis is a great mimicker of many diseases [5]. Diagnosis is usually presumptive because of variable presentation and difficulty in obtaining culture and histopathology tissues [6]. Diagnosis requires a high index of suspicion and is made by exclusion of other causes of uveitis like sarcoidosis and autoimmune diseases. The diagnosis of TbU can be confirmed by finding caseating granuloma, acid-fast bacilli on histopathology of ocular tissues and on isolation of the organism on AFB culture or by Nucleic acid amplification tests. Usually histopathologic specimens reveals a paucity of organisms [7]. The absence of pulmonary Tb does not rule out the possibility of ocular Tb, as majority of patients with extrapulmonary Tb have no evidence of active pulmonary TB [2, 8].

Tuberculous uveitis is treated with standard antituberculous therapy (ATT), 2 months of intensive phase followed by 10 months of continuous phase combined with systemic corticosteroids till clinical response is seen then tapered off [2]. There is paucity of data from our country on TbU outcomes likely because of underreporting, so this study was aimed to assess the effect of ATT in patients with TbU.

## Main text

### Methods

An observational prospective study was conducted on patients with TbU at JMCH Karachi, Pakistan from July to December 2017. JMCH is a 500 bed teaching hospital spread over 6 acres of land in Korangi, Karachi Pakistan. TbU patients were referred for treatment after TBU diagnosis by an ophthalmologist from Layton Rahmatullah Benevolent Trust (LRBT). LRBT is a charitable eye hospital and Pakistan’s largest non-governmental organization established in 1984. These patients presented at LRBT with complain of blurring of vision, photophobia, pain, redness, floaters or photophia. Patients with suspected of uveitis underwent detailed history, general and slit lamp examination. Then they were classified according to standardization of Uveitis Nomenclature (SUN) workshop [9] classification into anterior, Intermedia and posterior uveitis. TB uveitis was defined as an unexplained uveitis after thorough diagnostic workup, and/or ocular abnormalities compatible with TB, and/or abnormalities on chest radiology compatible with TB.

The patient were also inquired about history of fever, weight loss, history of Tb contact in family, previous history of Tb and associated symptoms. A detailed physical examination was done. All patients were investigated at baseline with chest xray, ESR and Montoux test (MT). Patients with TbU were started on ATT (usually 2 months of rifampicin, isoniazid, pyrizinamide, and ethambutol followed by months of rifampicin and isoniazid for total of 1 year duration). Clinical response was assessed by ophthalmologist at LRBT through slit lamp examination and visual acuity at 1, 3, 6 and 12 months followup. Informed consent was obtained from all patients or the attendant next of kin. JMCH ethical review committee approved study protocol before the commencement of the study. All data was collected on predesigned performa.

#### Statistical analysis

All analyses were conducted by using the SPSS (Release 19.0, standard version, copyright © SPSS; 1989–2002). A descriptive analysis was performed for demographic features presented as mean ± SD for quantitative variable that is age. Number (percentage) for qualitative variables that is, gender, history of TB, type of uveitis and outcome of uveitis that included change in inflation complete resolution and no change was done.

### Results

A total of 40 cases of TbU was referred to our center for treatment during the study time period. Mean age was 36 ± 3 years, 24 (60%) were female and 24 (60%) of patient had a family history of Tb. Blurring of vision and floaters was reported in 40 (100%) of patients followed by decrease vision and eye redness (Table 1). Patients with decrease vision had visual acuity of 6/18 and 6/36 at the start of treatment and at the end of treatment visual acuity corrected to 6/6 in all of these patients.

**Table 1:** Baseline characteristics of patients with TbU

Demographics	Frequency (n = 40)
Mean age	36 ± 3 years
Gender	
Male	16 (40%)
Female	24 (60%)
Previous history of TB*	12 (30%)
Family history of TB	24 (60%)
Montoux test (mm)	
< 15	14 (35%)
> 15	26 (65%)
ESR (mm)	
< 60	10 (25%)
> 60	30 (75%)
Symptom	
Redness of eye	24 (60%)
Eye pain	6 (15%)
Photophobia	12 (30%)
Blurred vision	40 (100%)
Dark, floating spots in field of vision (floaters)	40 (100%)
Decreased vision	24 (60%)

* Received prior ATT

Abnormal chest x-ray finding was present in 12 (30%) of patients, which included nodular infiltrates in 6 patients, upper lobe cavitation in 2 patients and 4 patients had bilateral hilar lymphadenopathy. Steroids were received by 3 (7.5%) patients where there was evidence of acute vitritis suggested by an ophthalmologist. Prednisolone was given in a dose of 1 mg/kg given for 3 weeks, then tapered off, according to the response to the treatment. We found posterior uveitis as the commonest form of TbU (90%). After taking 12 months of standard ATT 32 (80%) of patients had complete resolution of symptoms, 14% had partial resolution while the 2 (6%) had no improvement on treatment (Table 2).

**Table 2:** Type and outcome of Tb uveitis among study population

Type of uveitis	Percentage
Pan uveitis	10% (4)
Anterior uveitis	0%
Intermediate uveitis	0%
Posterior uveitis	90% (36)

Outcome	Frequency
Resolved	32 (80%)
Change in inflammation	6 (14%)
No change	2 (6%)

### Discussion

In this study, we found that posterior uveitis is the commonest TbU presentation in our patients. Overall, our patient population had a good clinical response and complete recovery on standard ATT. We found posterior uveitis in 90% of our patients, which is similar to the data from India [5, 10]. However, data from developed countries is different, a study from the Netherlands exclusively studied TbU and found Panuveitis in 51.5%, followed by posterior uveitis 30.3% [11]. Another study from the UK found chronic Panuveitis as commonest TbU [3] may be because of difference of population and regions.

Many patients also had no history of pulmonary or extrapulmonary Tb other than uveitis [2] as seen in our patients where only few patients had an abnormal Chest x ray which was found on routine work-up with no complaints of cough and sputum production. As Pakistan is a Tb endemic country we found that majority patient had a positive TB contact history, which can be an important clue in such patients when suspecting TbU as seen previously in a study from Iraq [12].

Tuberculin skin testing TST or MT was done in all patients and was found > 15 mm induration in the majority of patients of TbU. These results are similar to the study published from Iraq, where they also found significant high induration in ocular TB [13]. However, this testing has limitation in developing countries as MT is positive in the general population. Along with MT we also found higher ESR in our patients. Complete recovery seen in 80% of our patients compared to previous studies where the success rate was 70%, 70% and 60% respectively [3, 11, 14]. While all patients had favorable outcomes with no recurrence of disease in a study from Iraq [13]. Two of our patients had no response to treatment that may be because of underlying fibrosis or delayed in seeking medical treatment. Three of our patients received corticosteroids with signs of acute vitritis. The use of systemic steroids is controversial [15] they are usually added to treat inflammation and macular edema [16]. The posterior uveitis was seen commonly in our study followed by pan uveitis. This is reported to be commonest form of uveitis from India in TB uveitis [2, 5] while study from Saudia, Italian and Swiss center showed pan uveitis followed by posterior uveitis in presumed TbU [8, 17].

### Conclusion

In high burden countries, the TbU should be suspected among patients presenting with symptoms of blurring of vision, floaters, decrease vision and eye redness, treatment with ATT has a favorable outcome. It is important to diagnose TbU timely to prevent permanent damage and visual loss. Although it remains difficult to diagnose, early recognition of the correct diagnosis and specific therapy avoids recurrences, improves visual acuity and intra-ocular inflammation.

## Limitations

This study showed TbU experience in term of its treatment outcome. However, it has some limitations, (1) it’s a single center study, (2) limited number of patients, (3) there is always limitation in diagnosing TbU, diagnosis was based on history and examination while GenXpert testing was not done. Large multicenter studies are required to study further patient characteristics and clinical response in Tb endemic countries like Pakistan.

## Data Availability

All data generated or analysed during this study are included in this published article.

## Abbreviations

Tb: tuberculosis
TbU: tuberculous uveitis
ATT: anti-tuberculous
JMCH: Jinnah Medical College Hospital
LRBT: Layton Rahmatullah Benevolent Trust
SUN: standardization of Uveitis Nomenclature
MT: Montoux test
TST: tuberculin skin testing
